# A pilot study assessing the similarity between core outcome sets and outcomes included in health technology assessments

**DOI:** 10.12688/f1000research.73647.2

**Published:** 2022-02-28

**Authors:** Peter Cox, Paula R. Williamson, Susanna Dodd

**Affiliations:** 1 Institute of Systems, Molecular and Integrative Biology, University of Liverpool, Liverpool, UK; 2 Department of Health Data Science, University of Liverpool (a member of Liverpool Health Partners), Liverpool, UK

**Keywords:** Core outcome set, COS, health technology assessment, HTA, outcome research

## Abstract

**Objective: **Core outcome sets (COS) are an agreed standardised collection of outcomes created with representation from all key stakeholders (such as patients, clinicians, researchers), which should be reported as a minimum for all trials in that corresponding clinical area. There has been little research investigating the use of core outcomes in Health technology assessments (HTAs) and none in non-oncology HTAs. This study aimed to assess the similarity between COS and HTA outcomes.

**Methods: **Ten COS published between 2015 and 2019 were selected, with patient participation taken as a proxy measure for a high quality COS. The INAHTA database was used as a source to identify relevant HTAs, which were accessed through the hyperlinks provided. Outcomes selected for these assessments were categorised as either a specific, partial or no match compared to the COS. An additional cohort of non-oncology HTAs published between 2019 and 2021 were identified from the NICE website and compared against a relevant COS.

**Results:** Six hundred and fifty-one HTAs were matched to the ten COS areas, of which 119 were reviewed. Of a possible 1318 core outcome matches, there were 562 (43%) matches, 413 (31%) specific and 149 (11%) partial. NICE HTA matches against corresponding COS ranged from 44% to 100%, with a total of 78% (73/94) matches, 57 (61%) specific and 16 (17%) partial.

**Conclusion: **Further work is required to promote the awareness and implementation of COS within HTAs. The degree of matching between COS and NICE HTA outcomes is encouraging, demonstrating acceptance of COS by HTA producers.

## Introduction

Clinical trials are performed to evaluate the effects of treatment interventions, with the gold standard being randomised controlled trials. ^1^ Fundamentally this includes a test and a control treatment, with random assignment of treatment groups and at least one outcome measure. ^2^ Researchers decide on outcomes which best answer their research question, and consequently differences arise between trials in the same field as the chosen outcome measures are often of particular relevance to each study. The lack of uniformity amongst trial designs is abundant, demonstrated by a review of 8942 oncology trials which revealed that over 25,000 outcomes occurred only once or twice ^3^; furthermore the top cited and most accessed Cochrane reviews in 2009 described problems due to inconsistences in the reported outcomes in included studies. ^4^ Discrepancies are also observed when examining how outcomes are measured; for example, a survey of 10,000 trials investigating schizophrenia discovered 2194 different measurement scales were employed. ^5^ This produces a substantial challenge in data analysis, limiting the ability to compare studies, synthesise the available evidence and perform meta-analysis, ultimately leading to avoidable research waste. ^6^ There are also questions concerning whether trial outcomes are always relevant to patients or clinicians, meaning statistically significant results may have limited clinical bearing, hence not translating into improved clinical care. ^7^

In January 2010 the Core Outcome Measures in Effectiveness Trials (COMET) Initiative was launched with the aim of bringing together people interested in the development and application of core outcome sets (COS). COS address the issue of inconsistency and outcome reporting bias, while reducing the difficulties facing systematic reviewers due to heterogeneity in outcome measures. ^8^ COS are an agreed standardised collection of outcomes which should be reported as a minimum for all trials in the corresponding clinical area. The Core Outcome Set-STAndard for Development (COS-STAD) provides criteria against which to assess the quality of COS. ^9^ Researchers are not restricted to the COS, but there is an expectation it will always be reported. ^8^ The importance of COS has become increasingly acknowledged over time, with COMET endorsed by trial funders such as the National Institute for Health Research (NIHR), the Cochrane Collaboration and National Institute for Health and Care Excellence (NICE) . There is currently ongoing work assessing the representation of COS in US Food and Drug Administration (FDA) and European Medicines Agency (EMA) regulatory guidance. ^10^

Health technology assessments (HTAs) are multidisciplinary processes which use explicit methods to determine the value of a health technology at different points in its lifecycle. ^11^ They are produced to inform decision makers and promote an equitable, efficient and high-quality health system. ^11^ The technologies are often interventions designed to prevent, diagnose or treat medical conditions. The International Network of Agencies for Health Technology Assessments (INAHTA) is a network of 51 different HTA agencies which support healthcare decision making. This network produces the international HTA database, which provides a free, single point of access to information about ongoing and published HTAs.

Recent work has assessed the International Consortium for Health Outcomes Measurement (ICHOM) outcome sets against oncology HTAs, finding that HTAs tend to focus on generic measures which allow comparison across disease areas. However, it should be noted that these outcome sets are developed for routine care, unlike COS which are produced specifically for research so are more relevant for HTAs. ^12^ Additionally, a review of technology appraisal oncology scopes found that in the majority of cases there was complete overlap with those outcomes in COS; in a small number of exceptions the COS included two additional outcomes to those specified in the scope. ^13^ Other work has investigated a trial funder and member of INAHTA, the National Institute for Health Research Health Technology Assessment (NIHR HTA), examining their recommendation for applicants to search for a COS to include in their trial. By examining research applications and then surveying applicants it was found that 38% (36/95) searched for a COS through either the COMET database or another method, e.g. review of the literature; where a published COS existed it was included in 29% (7/24) of cases. ^14^ There is reason to believe that uptake may have increased, as a limitation was that no COS existed for 68 of the 95 studies assessed at the time of submission. ^14^ However, this study assessed the uptake of COS in trials rather than HTAs, and to our knowledge, ours is the first study to examine the similarity between COS for research and HTA outcomes. There is potential for COS to further improve the quality of HTAs, providing more uniform and patient-centred evidence to inform clinical guidelines and policy makers, such as NICE.

The purpose of this study was to assess the similarity between COS and outcomes included in HTAs.

## Methods

The COMET database, a database of studies relevant to the development of COS, was searched to select individual COS for this review. As a surrogate measure to ensure quality, only COS published between 2015 and 2019 where patients were included were selected, maximising the number of COS-STAD standards met. ^9^ The methodology of producing COS has improved with time, hence this 5-year period was chosen to include the most recent COS, which had a greater likelihood of including outcomes most relevant to all appropriate stakeholders. Additionally, COS developed for common diseases and interventions were selected to ensure sufficient HTAs were available for assessment. COS outcomes were extracted by one author (PC) from a pre-existing COMET database of outcomes from all COS for research published up to 2019.

The INAHTA database was searched in February 2021 for relevant HTAs, using the disease name as the search term with the English language filter applied. Where an intervention was specified by the COS this was included as a search term, e.g. obesity AND surgery. Quotation marks were used to refine searches that returned a large number of HTAs or when the results lacked specificity to the desired condition, e.g. searching type 1 diabetes produced HTAs relating to type 2 and gestational diabetes. There were no restrictions by year or country. MeSH terms were employed when there were variations in how search terms were spelt, for example postpartum haemorrhage versus postpartum hemorrhage.

HTAs were accessed using the hyperlinks provided in the INAHTA database. Where the hyperlink did not direct to the HTA but to the publisher’s website, this was searched using the title of the HTA. If a hyperlink did not work, the abstract in the INAHTA record was screened for outcomes if available. HTAs which were irrelevant to the COS, inaccessible (e.g. due to a non-functional hyperlink or no hyperlink was present) or which summarised clinical evidence with no outcome measures stated were excluded. Outcome measures were then extracted from the HTA alongside study identifier, year and data source. Data extraction involved creating a table comprising the core outcomes as headings with each HTA listed below. For each HTA, outcomes were matched to the appropriate headings and then colour coded green, yellow or red to indicate specific, partial or no match respectively. For partial matches, bold and italic text indicated an outcome more general or specific than the COS respectively.

Outcomes were categorised as either a specific match, a partial match or no match compared to the COS, following an approach used previously. ^15^^,^^16^ We considered a match between an HTA outcome and a core outcome to exist if they were either specifically or partially related. We defined a specific match as one where both outcomes corresponded to each other exactly, while a partial match was defined as one where the outcomes correspond to each other non-specifically. Taking the example of swollen joint count as a core outcome, if the HTA included swollen joint count as an outcome it would be considered a specific match, whilst disease activity would be considered a partial match. Partial matches were further categorised according to whether the HTA outcome was either more general or more specific than the COS outcome. The outcome matching process was quality checked by a seconder reviewer (SD). The data extracted from each HTA included the HTA identifier, publisher, publication year and outcome measures. Outcomes were ascertained from stated outcomes of interest or from the PICO (population, intervention, comparator, outcomes) statement. Where outcomes were not explicitly stated, the HTA was reviewed to elicit them and on occasion outcomes had to be ascertained from a series of key research questions. Outcomes identified in the scope of an HTA which could not be reported due to a lack of available data were included in the analysis. Considering the research question “what is the expected beneficial effect of atezolizumab on mortality?” as an example, this was deemed a specific match for overall survival and a partial match for cancer-specific survival.

Within the UK, NICE HTAs are of particular importance. It was noted that no NICE HTAs had been registered on the INAHTA database beyond 2011, so in April 2021, the NICE website was searched for HTAs corresponding to each of the COS from the original cohort. Non-oncology conditions were selected given previous work has examined uptake across oncology HTAs. ^12^^,^^13^ and where no appropriate NICE HTAs were identified, alternative COS were selected, using the same criteria of publication between 2015 and 2019 with patient involvement. The most recently published reports which matched a COS were included, with outcomes compared to core outcomes from the corresponding COS.

The results are presented through tables, graphs and using descriptive statistics. The analysis was performed using Excel 2019. Comparison between the INAHTA and NICE results was not examined given they cover different time periods and utilised different COS.

## Results

**Figure 1.**
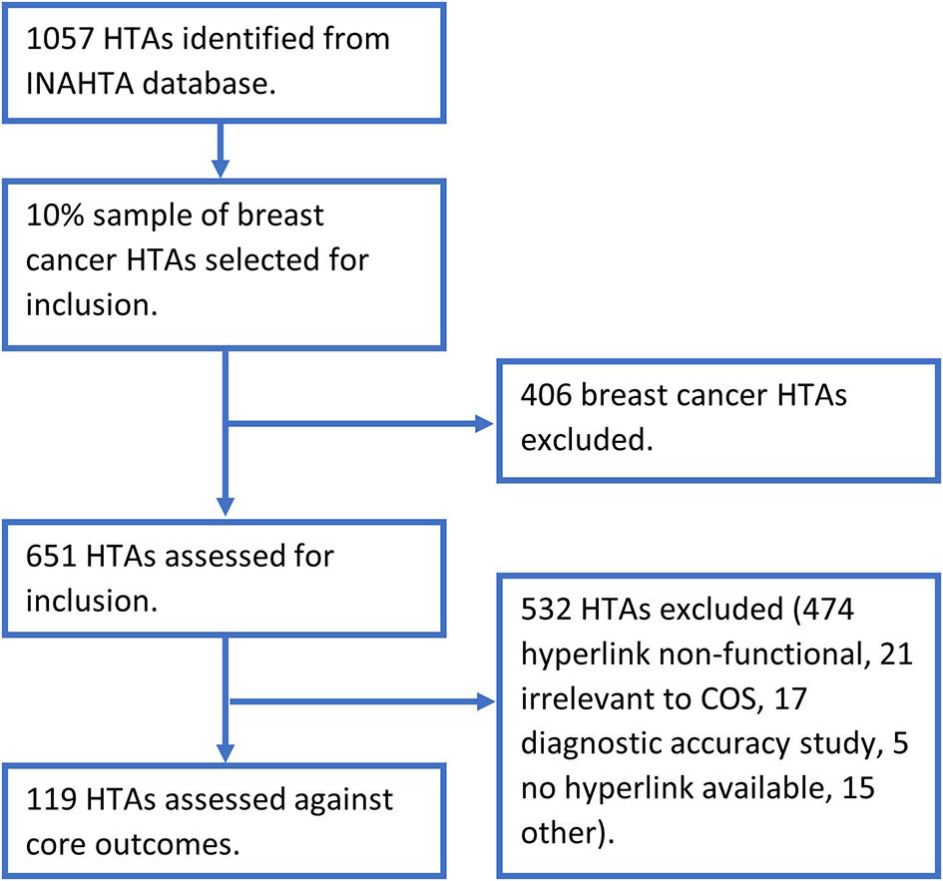
HTA identification and selection flowchart. COS, core outcome set; HTA, health technology assessment.

Ten COS were selected to assess against the INAHTA database. The search terms for each are displayed in Table 1. The search relating to the relapsing remitting multiple sclerosis and clinically isolated syndrome COS was performed as two individual searches to ensure all relevant HTAs were identified. The database search returned a cumulative total of 1057 HTAs, ranging from three for clinically isolated syndrome to 451 for breast cancer. A selection of 10% (45) of the breast cancer results were sampled, with the most recently published HTAs chosen as they were most likely to be accessible, detailed in Figure 1. This selection of HTAs comprised the most recently published HTAs until the 10% sample was reached, encompassing HTAs published between 2015 and 2020. This produced a possible 651 HTAs to be included in the report. Eight of the included COS applied to any intervention, with metabolic and bariatric surgery specified for obesity and treatment specified for postpartum haemorrhage. For each of the ten COS, the majority of the HTAs were not assessed as the hyperlinks listed in the INAHTA database were non-functional, detailed in Table 2. There were several HTAs irrelevant to the COS which were therefore excluded. Other reasons for exclusion included diagnostic accuracy study, HTA where no hyperlink was available, HTA not in English, HTA recommendations, HTA inaccessible on the source website, duplicates and where an updated version of the HTA had already been assessed. Systematic reviews provided over 90% of studies included, the remainder being trials. To minimise similarity to COS being underestimated by reviews, through outcome reporting being limited to what data exists in the literature, outcomes stated in the methods were included in the analysis, independent of the findings.

**Table 1.** Summary of search terms and number of search results.

COS	Intervention	Search term	Number of search results
Rheumatoid arthritis ^17^	Any	rheumatoid arthritis	135
Relapsing remitting Multiple sclerosis/Clinically isolated syndrome ^†^ ^18^	Any	relapsing remitting multiple sclerosis	36
		clinically isolated syndrome	3
Obesity ^19^	Metabolic and bariatric surgery	obesity AND surgery	62
Epilepsy ^20^	Any	epilepsy	91
Type 1 diabetes ^21^	Any	“type 1 diabetes”	67
Acne ^22^	Any	acne	7
Coronary artery disease ^23^	Any	coronary artery disease	134
Postpartum haemorrhage ^24^	Any treatment	“Postpartum Hemorrhage” [mh]	8
Psoriasis ^25^	Any	psoriasis	63
Breast cancer ^‡^ ^26^	Any	“breast cancer”	451

COS, core outcome set.

† The search relating to the relapsing remitting multiple sclerosis/clinically isolated syndrome COS was performed as two individual searches.

‡ 10% (45) of the breast cancer HTAs were assessed and included in the report.

**Table 2.** Breakdown of database search results for each individual COS.

Disease name	Number of search results	HTAs assessed	Hyperlink non-functional	Irrelevant to COS	Diagnostic accuracy study	No hyperlink available	Other
RA	135	22	106	4	3	0	0
MS/CIS	39	8	27	1	0	1	2
Obesity	62	10	52	0	0	0	0
Epilepsy	91	15	70	2	3	1	0
T1DM	67	19	41	2	0	0	5
Acne	7	1	5	1	0	0	0
CAD	134	17	104	4	7	0	2
PPH	8	2	2	3	0	0	1
Psoriasis	63	10	47	3	0	1	2
Breast cancer	451 ^†^	15	20	1	4	2	3
Total	651	119	474	21	17	5	15

Note: Other: Duplicates, HTA recommendation, HTA not in English, updated version of HTA included, HTA inaccessible on source website.

CAD, coronary artery disease; COS, core outcome set; HTA, health technology assessment; MS/CIS, multiple sclerosis/clinically isolated syndrome; PPH, postpartum haemorrhage; RA, rheumatoid arthritis; T1DM, type 1 diabetes mellitus.

† 10% (45) of the breast cancer HTAs were assessed and included in the report.

Overall, 119 HTAs were assessed against core outcomes with a maximum of 1318 matches possible, shown in Table 3. In total, there were 562 (43%) core outcome matches, 413 (31%) specific and 149 (11%) partial, with the percentage values rounded to whole numbers. Rheumatoid arthritis had the greatest percentage of specific matches (52%), postpartum haemorrhage returned the greatest percentage of partial matches (50%) while epilepsy and psoriasis both had the greatest percentage of no matches (67%). Only rheumatoid arthritis, type 1 diabetes, acne and postpartum haemorrhage had more total core outcome matches compared to no matches. The number of HTAs assessed ranged from one for acne to 22 for rheumatoid arthritis. The COS for rheumatoid arthritis had the greatest median value of matches with 69% while epilepsy had the fewest with a median of 25%, detailed in Table 4.

**Table 3.** The number of outcomes for each COS categorised as specific, partial or no match.

COS	Number of COs	Specific match (% of total)	Partial match (% of total)		No match (% of total)	Total
			More general	More specific		
Rheumatoid arthritis	8	91 (52)	16 (9)	1 (1)	68 (39)	176
Relapsing remitting Multiple sclerosis/Clinically isolated syndrome	9	28 (39)	1 (1)	2 (3)	41 (57)	72
Obesity	9	34 (38)	4 (4)	0 (0)	52 (58)	90
Epilepsy	8	36 (30)	1 (1)	3 (3)	80 (67)	120
Type 1 diabetes	8	57 (38)	19 (13)	1 (1)	75 (49)	152
Acne	7	3 (43)	0 (0)	1 (14)	3 (43)	7
Coronary artery disease	11	53 (28)	13 (7)	1 (1)	120 (64)	187
Postpartum haemorrhage	12	2 (8)	12 (50)	0 (0)	10 (42)	24
Psoriasis	10	30 (30)	3 (3)	0 (0)	67 (67)	100
Breast cancer	26	79 (20)	71 (18)	0 (0)	240 (62)	390
Total		413	140	9	756	1318

Note: Partial matches are further categorised according to whether the HTA outcome was either more general or more specific than the core outcome.

COS, core outcomes set; COs, core outcomes.

**Table 4.** Number of HTA outcomes with partial or specific match to core outcomes.

COS	Number of COs	Number of specific and partial matches to COs for each individual HTA (% of total)	Median %	% Range
RA	8	8 (100), 8 (100), 5 (63), 5 (63), 0 (0), 8 (100), 8 (100), 8 (100), 0 (0), 8 (100), 4 (50), 3 (38), 7 (88), 2 (25), 2 (25), 6 (75), 8 (100), 8 (100), 3 (38), 7 (88), 0 (0), 0 (0)	69	0, 100
MS/CIS	9	2 (22), 3 (33), 2 (22), 4 (44), 4 (44), 8 (89), 4 (44), 4 (44)	44	22, 89
Obesity	9	6 (67), 5 (56), 2 (22), 4 (44), 6 (67), 2 (22), 1 (11), 4 (44), 5 (56), 3 (33)	44	11, 67
Epilepsy	8	2 (25), 2 (25), 2 (25), 2 (25), 2 (25), 3 (38), 2 (25), 2 (25), 1 (13), 1 (13), 5 (63), 3 (38), 3 (38), 5 (63), 5 (63)	25	13, 65
T1DM	8	5 (63), 2 (25), 1 (13), 3 (38), 4 (50), 8 (100), 6 (75), 3 (38), 5 (63), 6 (75), 4 (50), 3 (38), 3 (38), 4 (50), 5 (63), 6 (75), 4 (50), 2 (25), 3 (38)	50	13, 100
Acne	7	4 (57)	57	n/a
CAD	11	4 (36), 7 (64), 6 (55), 9 (82), 6 (55), 0 (0), 0 (0), 5 (45), 7 (64), 4 (36), 0 (0), 4 (36), 5 (45), 0 (0), 1 (9), 4 (36), 5 (45)	36	0, 82
PPH	12	7 (58), 7 (58)	58	58, 58
Psoriasis	10	4 (40), 4 (40), 5 (50), 3 (30), 4 (40), 1 (10), 3 (30), 2 (20), 2 (20), 5 (50)	35	10, 50
Breast cancer	26	10 (38), 10 (38), 15 (58), 1 (4), 15 (58), 1 (4), 15 (58), 15 (58), 15 (58), 15 (58), 15 (58), 6 (23), 7 (27), 5 (19), 5 (19)	38	4, 58

CAD, coronary artery disease; COS, core outcome set; COs, core outcomes; HTA, health technology assessment; MS/CIS, multiple sclerosis/clinically isolated syndrome; PPH, postpartum haemorrhage; RA, rheumatoid arthritis; T1DM, type 1 diabetes mellitus.

To consider how the scope of the HTA compared to that of the COS, each HTA was assessed with regards to the investigated population and intervention. Table 5 shows the majority of the studies clustered around exact scope matches and the COS being broader, both in terms of the population and intervention.

**Table 5.** The scope of HTAs compared to the COS regards population and intervention.

		Intervention			
		COS is narrower	Exact match	COS is broader	Different intervention
Population	COS is narrower	2	10	11	0
	Exact match	1	22	23	0
	COS is broader	2	19	29	0
	Different subgroup of the population	0	0	0	0

COS, core outcome set.

**Figure 2.**
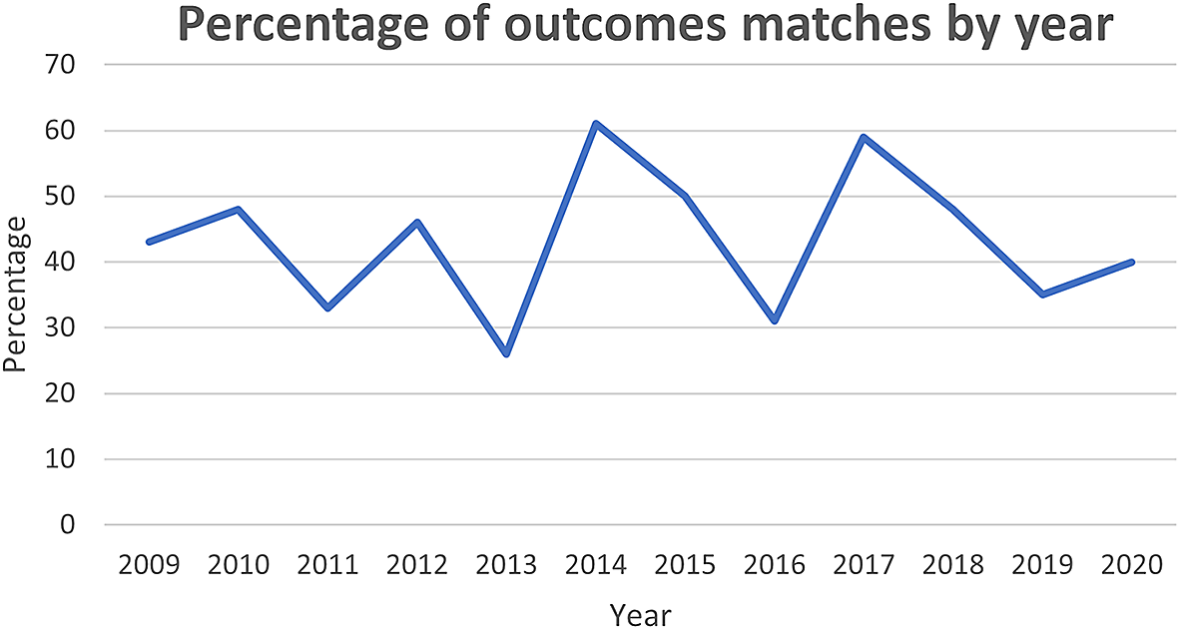
Percentage of outcome matches by year.

The distribution of HTAs by year ranged from 1998 to 2020, with 69% of included HTAs published in 2012 and beyond. The most HTAs published in a single year was 13 in 2012. A mean of nine HTAs were published per year in the following years between 2013 and 2020. Figure 2 shows from 2009 onward there has been some fluctuation in the degree of match, though ultimately the trend is stationary, remaining close to the mean of 43%. This may reflect the fact that, although the clinical trials community are becoming more aware of COS, they are yet to be adopted by the HTA community.

To assess the similarity between COS and recently published NICE HTA outcomes, the NICE website was searched for HTAs corresponding to each of the COS from the original cohort, except breast cancer. NICE HTAs were only identified for four COS, so six alternative COS were selected to include in the analysis. Ten non-oncology HTAs published between 2019 and 2021 were identified from the NICE website and compared against a relevant COS, shown in Table 6. HTA matches ranged from 44% to 100%, with a total of 78% (73/94) outcome matches with NICE HTAs, including 57 (61%) specific matches and 16 (17%) partial matches.

**Table 6.** Recently published non-oncology NICE HTAs compared with relevant COS, categorised as specific, partial or no match.

COS	Number of COs	Specific match (% of total)	Partial match (% of total)		No match (% of total)	HTA matches (% of total)
			More general	More specific		
Type 1 diabetes	8	4 (50)	1 (13)	0 (0)	3 (38)	5 (63)
Type 2 diabetes	18	16 (89)	1 (6)	0 (0)	1 (6)	17 (94)
Rheumatoid arthritis	8	8 (100)	0 (0)	0 (0)	0 (0)	8 (100)
Age-related macular degeneration	7	1 (14)	3 (43)	2 (29)	1 (14)	6 (86)
Multiple sclerosis (Relapsing remitting)	9	3 (33)	0 (0)	1 (11)	5 (56)	4 (44)
Multiple sclerosis (Secondary progressive)	8	4 (50)	0 (0)	0 (0)	4 (50)	4 (50)
Dravet syndrome	5	1 (20)	1 (20)	1 (20)	2 (40)	3 (60)
Psoriasis	10	5 (50)	0 (0)	0 (0)	5 (50)	5 (50)
Coronary artery disease	13	13 (100)	0 (0)	0 (0)	0 (0)	13 (100)
Crohn's disease	8	2 (25)	1 (13)	5 (63)	0 (0)	8 (100)
Total	94	57	7	9	21	73

Note: Partial matches are further categorised according to whether the NICE outcome was either more general or more specific than the core outcome.

COS, core outcome set; COs, core outcomes; HTA, health technology assessment.

## Discussion

This review found that HTAs in the INAHTA database included 43% of core outcomes from COS, with specific matches accounting for 31% and partial matches for 11%.

### Similarity between COS and HTA outcomes

This is a novel piece of work as there are few studies conducted assessing COS uptake, and none investigating their similarity with non-oncology HTA outcomes. Recent work has found that the uptake of COS in randomised controlled trials and systematic reviews varies greatly between different areas of health. ^27^ Barriers to COS uptake are also noted, with lack of awareness, lack of validated measures or no consensus on measures, and lack of patient involvement being the most common reasons reported. ^27^ Ideally with time, awareness will continue to grow, leading to the development of more high-quality COS which involve all key stakeholders including patients. The issue regarding a lack of validated measures, however, is a pertinent problem and one which goes hand in hand with the fundamentals of COS. Research to determine the most suitable outcomes to measure must be accompanied by work to determine how best to measure those outcomes. This is particularly important for life impact domains which tend to be patient reported. There are many well established methods to measure outcomes from clinical, resource use and adverse event domains as these are often linked to everyday work of professionals in healthcare, unlike life impact outcomes, the importance of which are becoming increasingly recognised as a result of COS.

A potential reason for reduced similarity between COS and HTA outcomes in this review may be the target populations selected when originally designing the COS. Eight of the ten COS related to any intervention for their given disease. The result is that studies investigating specific interventions, such bioabsorbable stents for coronary artery disease, are compared against measures designed for all coronary artery disease patients. A study investigating bioabsorbable stents may not have had the resources available to follow up their participants after five years to survey for outcomes such as stroke, depression or functional status, as this would have large time implications and ultimately may be of little relevance to their research question. Table 5 illustrates this point, with the distribution of HTAs around that of “COS is broader” for both population and intervention. Likewise, as many of the COS related to any individual with the disease, studies concerning specific populations would have fallen under their scope. While this could suggest that more COS are needed to cover the different subgroups, the value of COS are the uniformity they provide. If trials concerning subpopulations, such as pregnancy, advanced disease, or hormone responsive disease, recorded the outcomes designed for the greater population, it could reveal currently unknown trends, such as interventions experiencing improved efficacy in specific groups.

There are instances where outcomes identified in the scope of an HTA cannot be included in the final assessment as sufficient available data is lacking. This may reflect appreciation and acknowledgement of the importance of these outcomes by those appraising the value of a technology which needs to be filtered down to those designing and conducting trials. Trial funders and researchers may need to consider the time taken to design, conduct and report research to build a sufficiently detailed literature base. This would suggest value in allowing an extended period of time before further research to allow publication of studies which have incorporated COS into their protocols.

Another potential explanation for low representation of core outcomes could relate to the selection of COS published between 2015 and 2019. This was used as a proxy measure for quality COS, but the majority of the HTAs assessed (71/119) were published before 2015. While this could account for the lack of overlap prior to this year, Figure 2 demonstrates there has been no sustained increase in the percentage of outcomes included in HTAs.

As a 10% subset of the most recent breast cancer HTAs were included in the analysis, it is intriguing to note it is amongst the lowest matching conditions. While reports published from 2016 onwards were solely analysed, it should be noted that the COS included 26 separate core outcomes. This was markedly greater compared to other COS in the analysis and may point to the importance of being selective when developing COS, as trial designers may find vast numbers of outcomes too overwhelming to take them all into consideration. Including too many outcomes may also serve to dilute the relevance of other more significant outcomes.

Recent work has compared ICHOM standard sets to oncology HTAs using a qualitative approach. They found that HTAs favour more generic outcome measures which allow comparison with other disease areas, rather than disease-specific outcomes recommended by the ICHOM standard sets. ^12^ It has been shown that HTAs consider overall survival data to be most crucial when making decisions on the value of the technology. ^28^ However, overall survival data are not always mature when submitted for HTA assessment and so intermediate outcomes, such as progression-free survival, may be suitable for HTA agencies. HTA agencies only accept validated intermediate outcomes and the level of validity varies between HTA agencies. ^12^^,^^28^ However, it should be noted the study only focussed on five areas of oncology and that ICHOM standard sets are developed for routine care, whereas COS designed for research are therefore of more relevance for HTA agencies.

### INAHTA database

Despite the accessibility difficulties, the INAHTA database remains a great resource for identifying HTAs. It can be easily navigated and continues to update details of new HTAs whenever they become available. In response to feedback on the difficulties accessing the HTAs, the managers of the database recognised the hyperlink issues, which arose as the records are self-administered and the producer of the report adds their own copy, noting that the correction of this issue will take time.

A recent study surveying members of the INAHTA found that one of their biggest challenges is including stakeholders in HTAs, particularly where short deadlines offer little time for input. ^29^ Implementing COS that have been developed with patient input could ensure HTAs included patient representation and reduce the time-consuming process of surveying patients themselves.

### NICE HTAs

The finding that 78% of core outcomes were covered by NICE HTAs is very encouraging and notably greater than that for HTAs from the INAHTA database. This may be linked to the endorsement of COS by NICE or their rigorous approach of protocol design and stakeholder involvement.

### Limitations

The main limitation in this review was the large number of hyperlinks (474/651, 73%) in the INAHTA database which did not lead to a report, resulting in a large number of exclusions. This allowed for feedback to the database providers, beginning the process of amending the accessibility of the reports so future research can make use of the resource. On occasion the hyperlink led to the source website from which the HTA could be searched for, but this did not always produce the desired HTA. Additionally, only one database was searched for HTAs to include in the review potentially omitting appropriate HTAs not registered on the INAHTA database. Another limitation is that many of the COS included in the review related to any intervention for their given disease. This may have exaggerated reduced incorporation of core outcomes in the context of very specific HTAs.

Although the HTA outcome data extraction was conducted by a single researcher (PC), the opinion of a second researcher (SD) with extensive experience of outcome extraction and classification was consulted in all cases of doubt or ambiguity. In addition, all outcome matching assessments were double checked by this reviewer (SD).

## Conclusion

This novel piece of research includes, to our knowledge, the first comparison of non-oncology HTAs and COS for research. This pilot study highlights that further work is needed to promote awareness and implementation of COS within HTAs, with less than half of core outcomes currently measured. The degree of matching between COS and NICE HTA outcomes, albeit from a small sample, suggests this is both practical and achievable with the additional benefit of increasing patient representation, a particular challenge for HTA agencies. Incorporation of COS will allow HTA findings to better reflect the opinions and preferences of all relevant stakeholders.

## Data availability statement

### Underlying data

The COMET database was utilised to identify suitable COS. Each of the 10 COS are accessible though the following hyperlinks: rheumatoid arthritis, relapsing remitting multiple sclerosis/clinically isolated syndrome, obesity, epilepsy, type 1 diabetes, acne, coronary artery disease, postpartum haemorrhage, psoriasis, breast cancer. HTAs can be accessed through the INAHTA database. The search terms are described in Table 1.

The 10 COS compared to the NICE HTAs can be accessed through the following hyperlinks: type 1 diabetes, type 2 diabetes, rheumatoid arthritis, age-related macular degeneration, relapsing remitting multiple sclerosis/clinically isolated syndrome, secondary progressive multiple sclerosis, Dravet syndrome, psoriasis, coronary artery disease, Crohn’s disease.

The corresponding NICE HTAs can be accessed through their website or through the following hyperlinks: type 1 diabetes, type 2 diabetes, rheumatoid arthritis, age-related macular degeneration, relapsing remitting multiple sclerosis/clinically isolated syndrome, secondary progressive multiple sclerosis, Dravet syndrome, psoriasis, coronary artery disease, Crohn’s disease.

### Extended data

Harvard Dataverse: Data analysis for “A pilot study assessing the uptake of core outcome sets in health technology assessments” https://doi.org/10.7910/DVN/62NTAA.

Data are available under the terms of the Creative Commons Zero “No rights reserved” data waiver (CC0 1.0 Public domain dedication).
