# Supplementary material for: A pilot study assessing the similarity between core outcome sets and outcomes included in health technology assessments
Source: F1000Res. 2022 May 3;10:1084. Originally published 2021 Oct 25. [Version 3] doi: 10.12688/f1000research.73647.3 (PMC9069171; doi:10.12688/f1000research.73647.3)
Supplement: Peer review history

# Peer review: Reviewer response for version 3

**DOI:** 10.5256/f1000research.133578.r136562

**Published:** 2022-05-04

**Authors:** Alison E. Turnbull

**Affiliations:** 1 Division of Pulmonary and Critical Care Medicine, School of Medicine, Johns Hopkins University, Baltimore, MD, USA; 2 Department of Epidemiology, Bloomberg School of Public Health, Johns Hopkins University, Baltimore, MD, USA

The authors responded satisfactorily to my suggested revisions.  I have no further comments.

Is the work clearly and accurately presented and does it cite the current literature?

Yes

If applicable, is the statistical analysis and its interpretation appropriate?

Yes

Are all the source data underlying the results available to ensure full reproducibility?

Yes

Is the study design appropriate and is the work technically sound?

Yes

Are the conclusions drawn adequately supported by the results?

Partly

Are sufficient details of methods and analysis provided to allow replication by others?

Yes

Reviewer Expertise:

Clinical epidemiology, critical care outcomes, patient-centered care

I confirm that I have read this submission and believe that I have an appropriate level of expertise to confirm that it is of an acceptable scientific standard.

# Peer review: Reviewer response for version 2

**DOI:** 10.5256/f1000research.121621.r129869

**Published:** 2022-04-19

**Authors:** Alison E. Turnbull

**Affiliations:** 1 Division of Pulmonary and Critical Care Medicine, School of Medicine, Johns Hopkins University, Baltimore, MD, USA; 2 Department of Epidemiology, Bloomberg School of Public Health, Johns Hopkins University, Baltimore, MD, USA

This manuscript describes the similarity between outcomes used to evaluate health technologies and the outcomes included in Core Outcomes Sets (COS). Attempts to evaluate the effects of health technologies - termed health technology assessments (HTAs) - were identified through the International Network of Agencies for Health Technology Assessments (INHATA) and the National Institute for Health and Care Excellence (NICE) website. 90% of identified HTAs were systematic reviews and the remainder were trials. Ten COS that were developed for common diseases in collaboration with patients, and published between 2015 and 2019 were identified from the COMET database. Basic descriptive statistics were used to describe the similarity between outcomes in HTAs and each COS, to compare the populations and interventions targeted by COS and HTAs, and to describe changes in the level of similarity between COS and HTAs over time. The study's primary limitation, which the authors acknowledge, is that the vast majority of HTAs eligible for inclusion in the study did not have functional hyperlinks in INHATA, and it is unclear to what extent the included studies are representative of the entire target population of HTAs. Put another way, the external validity of the HTAs described is unknown.  

 My primary recommendation regards how the study's results have been interpreted. Conclusions in both the abstract and body of the manuscript address awareness, acceptance, and implementation of COS by the people designing HTAs. However, the temporal relationship between the publication of COS and the design of HTAs included in this study is not straightforward. Fully 60% of included HTAs were published before 2015 (and possibly designed years before publication), while the 10 COS were published between in 2015 and later. In Figure 2, there does not appear to be a temporal trend between 2009 and 2020 in the degree of similarity between COS and HTA outcomes. Thus, another interpretation of these results is that COS developers consistently include 40 - 70% of the outcomes assessed by HTAs when developing their COS and this does not appear to be changing. I recommend performing a sub-group analysis of HTAs developed after the publication of relevant COS to address this chicken-and-egg problem. The results of such an analysis may permit stronger and more substantiated inferences about the awareness, acceptance, and implementation of COS.

Is the work clearly and accurately presented and does it cite the current literature?

Yes

If applicable, is the statistical analysis and its interpretation appropriate?

Yes

Are all the source data underlying the results available to ensure full reproducibility?

Yes

Is the study design appropriate and is the work technically sound?

Yes

Are the conclusions drawn adequately supported by the results?

Partly

Are sufficient details of methods and analysis provided to allow replication by others?

Yes

Reviewer Expertise:

Clinical epidemiology, critical care outcomes, patient-centered care

I confirm that I have read this submission and believe that I have an appropriate level of expertise to confirm that it is of an acceptable scientific standard, however I have significant reservations, as outlined above.

# Response

**Published:** 2022-04-27

**Authors:** Peter Cox

**Affiliations:** University of Liverpool, UK

Dear Dr Turnbull

 Thank you for reviewing our manuscript “A pilot study assessing the similarity between core outcome sets and outcomes included in health technology assessments”. Thank you for your recommendation of a subgroup analysis, which we have undertaken. We have included the following paragraph to the results section alongside a new figure describing the percentage of outcome matches by year for HTAs developed after COS publication.

 “A drawback of these results is that many HTAs were produced prior to publication of the COS. To establish a clearer view on the impact of COS, a subgroup analysis consisting of HTAs developed following publication of the COS was undertaken. This comprised 25 HTAs across 8 of the selected COS. From a possible 373 there were 163 outcome matches, encompassing 109 specific matches and 54 partial matches. Accordingly 44% of HTA outcomes were covered by the COS, ranging from 52% in 2016 to 41% in 2019, with a trend of marginal decrease illustrated in Figure 3. This may reflect the fact that, although the clinical trials community are becoming more aware of COS, they are yet to be adopted by the HTA community.”

# Peer review: Reviewer response for version 2

**DOI:** 10.5256/f1000research.121621.r125645

**Published:** 2022-03-04

**Authors:** Catriona McDaid

**Affiliations:** 1 York Trials Unit, Department of Health Sciences, University of York, York, UK

We thank the authors for their detailed response to our comments and associated amendments. We have no further comments.

Is the work clearly and accurately presented and does it cite the current literature?

Yes

If applicable, is the statistical analysis and its interpretation appropriate?

Yes

Are all the source data underlying the results available to ensure full reproducibility?

Yes

Is the study design appropriate and is the work technically sound?

Yes

Are the conclusions drawn adequately supported by the results?

Partly

Are sufficient details of methods and analysis provided to allow replication by others?

Partly

Reviewer Expertise:

Applied health research, systematic reviews, randomised controlled trials, health technology assessment

I confirm that I have read this submission and believe that I have an appropriate level of expertise to confirm that it is of an acceptable scientific standard.

# Peer review: Reviewer response for version 1

**DOI:** 10.5256/f1000research.77311.r118406

**Published:** 2022-01-18

**Authors:** Catriona McDaid, Andrew Mott

**Affiliations:** 1 York Trials Unit, Department of Health Sciences, University of York, York, UK; 2 University of York, UK, York, UK

This peer review has been undertaken jointly with Andrew Mott, NIHR Pre-doctoral Research Fellow at the University of York. We each reviewed independently and then combined our reviews.

 The article explores the extent of agreement/match between 10 individual COS published between 2015 and 2019 and HTA reports published on the INHATA database up to February 2021, supplemented by a comparison between COS and a cohort of UK NICE HTA reports. Overall, the study methods and results are comprehensively reported, tables are informative, and the data underlying the results are available. There is no reference to a publicly available protocol, though we have not identified any obvious sources of potential bias. Key limitations are addressed. There are a few areas where the reporting could be made clearer and some aspects of interpretation given further consideration.

- The study is described as a pilot in the title and the conclusions. In the discussion there is also reference to “the feasibility assessment in this pilot study”. However, this is not apparent from the study aim/purpose or the methods so we suggest the term pilot is not used unless further information can be presented to show that it was indeed intended as a pilot – in which case the objective and methods would need amended to reflect this.
- We were not totally convinced by the rationale for excluding oncology studies from the NICE cohort but not the INHATA cohort, when the stated purpose of including the former was to fill the gap identified in what was recorded on the INHATA database. It also looks as though a different process was used – as we interpret it, for the INHATA part of the study the COS were identified first then the reports and vice versa for the NICE cohort. We don’t think this will have introduced any important bias though possibly you could end up with the UK reports covering a different time period. You could consider adding some further descriptive information on the NICE cohort on range of publication year as you have for the INHATA cohort so that the similarity of the two datasets is clear.
- Selecting a subset of 10% of the breast cancer HTAs seems reasonable given the volume. Given that the most recent were selected it would be worth picking up in the discussion what effect this might have on the results as these are more likely to have been undertaken after the COS were published than any of the other conditions. This might be of particular interest as it appears to be amongst the lower matching (median 38%) conditions.
- The methods state that the HTAs were taken from the INAHTA database. This database includes a variety of study designs and the term HTA can be defined in different ways. As reviewers we both interpreted differently whether or not trials were included so it would be useful to be explicit about what study designs were included under the term HTA. If all study designs were included some discussion around how the different designs may have affected the use of COS should be added.
- If available, the date of searching the NICE website should be added as the content is regularly changing.
- The methods state that COS were selected from the COMET website based on year of publication (2015-2019) and whether they addressed a common disease or intervention. Is there any further information on how the decision about being common was applied? A study selection flow diagram would also be a helpful addition to the text in the first paragraph of the results.
- Further information in the methods on how outcomes were identified/established/extracted from reports would be useful. There may not be full agreement between the outcomes of interest identified in the scope/methods of a technology assessment (defining this as evidence synthesis and cost-effectiveness analysis) and the outcomes that are eventually included in the assessment due to the lack of data on the specific outcome; for example an outcome may be defined as of interest in the scope but no relevant data found and included in the synthesis. Also in the scope the outcomes may be more broadly expressed but in the included studies outcomes may be more specific reflecting what they could find. How was this approached in the data extraction? This may seem pedantic/theoretical but is there a possibility that the scope/methods sections could match well to a COS if it is a recent report, but the outcomes reported in the actual included studies may not if the data available are mainly older trials. So, there is another time lag issue here. Some further unpacking of this may be worth considering and whether there are any related recommendations to be made regarding future research on this topic. To ensure study replicability some additional information in the methods on how data were extracted would be helpful.
- As the COSs were chosen initially and the HTAs identified that fit the COS it is possible these are not the most appropriate COS for each individual HTA. This is partially demonstrated in table 5 where almost half the HTAs have a narrower scope than the COS. It would be beneficial to have some discussion about whether these HTAs outcomes are specific to the narrower population or intervention.
- We agree with the authors that a limitation of the study is that the majority of the included reports were published prior the introduction of the COS. In some parts of the paper e.g., the title and some parts of the discussion, we are not sure that the terminology ‘COS uptake’ is appropriate given that the COS was not necessarily available at the time a report was published. The terminology used for the study purpose and elsewhere “similarity between COS and outcomes included in HTAs” is more accurate and appropriate and this could be applied across the paper.
- The conclusion refers to “improved uptake across NICE HTAs” – we are not convinced that the data presented warrant such a strong conclusion.
- Underlying data – For the INHATA database only the general link to the site is available – is it possible to provide a list of the included reports?

Is the work clearly and accurately presented and does it cite the current literature?

Yes

If applicable, is the statistical analysis and its interpretation appropriate?

Yes

Are all the source data underlying the results available to ensure full reproducibility?

Yes

Is the study design appropriate and is the work technically sound?

Yes

Are the conclusions drawn adequately supported by the results?

Partly

Are sufficient details of methods and analysis provided to allow replication by others?

Partly

Reviewer Expertise:

Applied health research, systematic reviews, randomised controlled trials, health technology assessment

We confirm that we have read this submission and believe that we have an appropriate level of expertise to confirm that it is of an acceptable scientific standard, however we have significant reservations, as outlined above.

# Response

**Published:** 2022-02-22

**Authors:** Peter Cox

**Affiliations:** University of Liverpool, UK

Dear Dr McDaid and Mr Mott,

 Thank you for reviewing our manuscript “A pilot study assessing the uptake of core outcome sets in health technology assessments”. We hope that the corrections we have made capture your points.

 Yours sincerely,

 Peter Cox

**The study is described as a pilot in the title and the conclusions. In the discussion there is also reference to “the feasibility assessment in this pilot study”. However, this is not apparent from the study aim/purpose or the methods so we suggest the term pilot is not used unless further information can be presented to show that it was indeed intended as a pilot – in which case the objective and methods would need amended to reflect this.**

**Author response:** *Thank you for raising this point. This study was conceptualised as a small scale study investigating the similarity between COS and HTA outcomes, given the presence of few prior studies assessing COS uptake. The results of this study provide backing for a similar study on a larger scale, to assess if these trends identified hold true given a larger sample size. This led to the description as a pilot study.*

*The following line regards the feasibility assessment has been removed, “This was an important finding in terms of the feasibility assessment in this pilot study”.*

**We were not totally convinced by the rationale for excluding oncology studies from the NICEcohort but not the INHATA cohort, when the stated purpose of including the former was tofill the gap identified in what was recorded on the INHATA database. It also looks as though a different process was used – as we interpret it, for the INHATA part of the study the COS were identified first then the reports and vice versa for the NICE cohort. We don’t think this will have introduced any important bias though possibly you could end up with the UK reports covering a different time period. You could consider adding some further descriptive information on the NICE cohort on range of publication year as you have for the INHATA cohort so that the similarity of the two datasets is clear.**

**Author response:** *Thank you for this comment. NICE HTAs are of particular importance in the UK and following the discovery recent HTAs were not registered on the INAHTA database beyond 2011, they were searched for and identified from the NICE website to supplement our findings. Given COS vs oncology NICE HTAs has already been assessed in a previous project [Kalf et al.], non-oncology COS were assessed. From the original cohort of 10 COS, relevant NICE HTAs were only identified for 4 COS. Subsequently a further 6 non-oncology COS were selected to be assessed against NICE HTAs, providing the results in table 6.*

*The methods text has been edited to include the following:*

*“Within the UK, NICE HTAs are of particular importance. It was noted that no NICE HTAs had been registered on the INAHTA database beyond 2011, so in April 2021, the NICE website was searched for HTAs corresponding to each of the COS from the original cohort. Non-oncology conditions were selected given previous work has examined uptake across oncology HTAs* *(12, 13)* *and where no appropriate NICE HTAs were identified, alternative COS were selected, using the same criteria of publication between 2015 and 2019 with patient involvement. The most recently published reports which matched a COS were included, with outcomes compared to core outcomes from the corresponding COS.*

*“The results are presented through tables, graphs and using descriptive statistics. The analysis was performed using Excel 2019. Comparison between the INAHTA and NICE results was not examined given they cover different time periods and utilised different COS.”*

*The results section has been edited to include the following:*

*“To assess the similarity between COS and recently published NICE HTA outcomes, the NICE website was searched for HTAs corresponding to each of the COS from the original cohort, except breast cancer. NICE HTAs were only identified for four COS, so six alternative COS were selected to include in the analysis. Ten non-oncology HTAs published between 2019 and 2021 were identified from the NICE website and compared against a relevant COS, shown in Table 6.”*

**Selecting a subset of 10% of the breast cancer HTAs seems reasonable given the volume.Given that the most recent were selected it would be worth picking up in the discussionwhat effect this might have on the results as these are more likely to have been undertakenafter the COS were published than any of the other conditions. This might be of particular interest as it appears to be amongst the lower matching (median 38%) conditions.**

**Author response:** *Thank you for this consideration. The following has been added to the discussion stating the dates of the HTAs included and to suggest why this COS had amongst the lowest outcome matching.*

*“As a 10% subset of the most recent breast cancer HTAs were included in the analysis, it is intriguing to note it is amongst the lowest matching conditions. While reports published from 2016 onwards were solely analysed, it should be noted that the COS included 26 separate core outcomes. This was markedly greater compared to other COS in the analysis and may point to the importance of being selective when developing COS, as trial designers may find vast numbers of outcomes too overwhelming to take them all into consideration. Including too many outcomes may also serve to dilute the relevance of other more significant outcomes.”*

**The methods state that the HTAs were taken from the INAHTA database. This database**

**includes a variety of study designs and the term HTA can be defined in different ways. As reviewers we both interpreted differently whether or not trials were included so it would be useful to be explicit about what study designs were included under the term HTA. If all study designs were included some discussion around how the different designs may have affected the use of COS should be added.**

**Author response:** *Thank you for this comment. The following has been added to the results section to clarify the different study designs included in the analysis.*

*“Systematic reviews provided over 90% of studies included, the remainder being trials. To minimise similarity to COS being underestimated by reviews, through outcome reporting being limited to what data exists in the literature, outcomes stated in the methods were included in the analysis, independent of the findings.”*

**If available, the date of searching the NICE website should be added as the content is**

**regularly changing.**

**Author response:** *The NICE website was searched in April 2021, this has been included in the methods section*.

**The methods state that COS were selected from the COMET website based on year of**

**publication (2015-2019) and whether they addressed a common disease or intervention. Is there any further information on how the decision about being common was applied? A**

**study selection flow diagram would also be a helpful addition to the text in the first**

**paragraph of the results.**

**Author response:** *A flow chart has been added to the beginning of the results and the methods section edited to include the following line:*

*“The selected COS were chosen as they were considered to be common conditions; examples of those excluded were: systemic sclerosis, malaria, thyroid eye disease, Hirschsprung's disease and twin–twin transfusion syndrome. This consideration heightened the likelihood sufficient HTAs were available for assessment”*

**Further information in the methods on how outcomes were identified/established/extracted**

**from reports would be useful. There may not be full agreement between the outcomes of interest identified in the scope/methods of a technology assessment (defining this as**

**evidence synthesis and cost-effectiveness analysis) and the outcomes that are eventually included in the assessment due to the lack of data on the specific outcome; for example an outcome may be defined as of interest in the scope but no relevant data found and included in the synthesis. Also in the scope the outcomes may be more broadly expressed but in the included studies outcomes may be more specific reflecting what they could find. How was this approached in the data extraction? This may seem pedantic/theoretical but is there a possibility that the scope/methods sections could match well to a COS if it is a recent report, but the outcomes reported in the actual included studies may not if the data available are mainly older trials. So, there is another time lag issue here. Some further unpacking of this may be worth considering and whether there are any related recommendations to be made regarding future research on this topic. To ensure study replicability some additional information in the methods on how data were extracted would be helpful.**

**Author response:** *Thank you for this consideration. Outcomes which were listed in the PICO or as a list of key research questions or equivalent were included and categorised as matches in our analysis. We have added the following line to the methods to clarify this and the paragraph to the discussion to give thought to the relevance of this:*

*“Outcomes identified in the scope of an HTA which could not be reported due to a lack of available data were included in the analysis.”*

*“There are instances where outcomes identified in the scope of an HTA cannot be included in the final assessment as sufficient available data is lacking. This may reflect appreciation and acknowledgement of the importance of these outcomes by those appraising the value of a technology which needs to be filtered down to those designing and conducting trials. Trial funders and researchers may need to consider the time taken to design, conduct and report research to build a sufficiently detailed literature base. This would suggest value in allowing an extended period of time before further research to allow publication of studies which have incorporated COS into their protocols.”*

**As the COS were chosen initially and the HTAs identified that fit the COS it is possible these are not the most appropriate COS for each individual HTA. This is partially demonstrated in table 5 where almost half the HTAs have a narrower scope than the COS. It would be beneficial to have some discussion about whether these HTAs outcomes are specific to the narrower population or intervention.**

**Author response:** *Thank you for this comment. The following has been included in the discussion addressing the scope of COS and relevance to narrower HTAs.*

*“Likewise [to COS being broader than many of the HTAs], as many of the COS related to any individual with the disease, studies concerning specific populations would have fallen under their scope. While this could suggest that more COS are needed to cover the different subgroups, the value of COS are the uniformity they provide. If trials concerning subpopulations, such as pregnancy, advanced disease, or hormone responsive disease, recorded the outcomes designed for the greater population, it could reveal currently unknown trends, such as interventions experiencing improved efficacy in specific groups.”*

**We agree with the authors that a limitation of the study is that the majority of the included reports were published prior the introduction of the COS. In some parts of the paper e.g., the title and some parts of the discussion, we are not sure that the terminology ‘COS uptake’ is appropriate given that the COS was not necessarily available at the time a report was published. The terminology used for the study purpose and elsewhere “similarity between COS and outcomes included in HTAs” is more accurate and appropriate and this could be applied across the paper.**

**Author response:** *Thank you for this comment and suggestion. To reflect this point COS uptake has been replaced throughout the text with “similarity between COS and HTA outcomes” when referring to our findings.*

*The title has been edited to “A pilot study assessing the similarity between core outcome sets and outcomes included in health technology assessments”*

**The conclusion refers to “improved uptake across NICE HTAs” – we are not convinced that the data presented warrant such a strong conclusion.**

**Author response:** *The conclusion has been edited to say “The degree of matching between COS and NICE HTA outcomes, albeit from a small sample, suggests this is both practical and achievable with the additional benefit of increasing patient representation, a particular challenge for HTA agencies”*

**Underlying data – For the INHATA database only the general link to the site is available – is it possible to provide a list of the included reports?**

**Author response:** *Links to individual HTA reports have been included in the data analysis on the Harvard Dataverse Repository under the extended data*.
